# Supplementary material for: Differential requirement for BRCA1-BARD1 E3 ubiquitin ligase activity in DNA damage repair and meiosis in the Caenorhabditis elegans germ line
Source: PLoS Genet. 2023 Jan 30;19(1):e1010457. doi: 10.1371/journal.pgen.1010457 (PMC9910797; doi:10.1371/journal.pgen.1010457)
Supplement: S1 Table — (DOCX) [file pgen.1010457.s001.docx]

**Supplemental Table 1: Strains**

| Strain | Genotype | Source |
| --- | --- | --- |
| N2 | Wild type | CGC |
| JEL1184 | *brc-1(xoe60[I23A])* | This study |
| JEL1200 | *brc-1(xoe62[triA])* | This study |
| JEL730 | *brc-1(xoe4[null])* | [1] |
| JEL1299 | *brc-1(xoe67[I59A, R61A])* | This study |
| NSV49 | *brc-1[ddr6(brc-1::HA])* | [2] |
| JEL1295 | *brc-1(xoe62[triA])::HA* | This study |
| CA324 | *zim-1(tm1813)* | [3] |
| JEL1215 | *brc-1(xoe60[I23A]); zim-1(tm1813)* | This study |
| JEL1221 | *brc-1(xoe62[triA]); zim-1(tm1813)* | This study |
| JEL749 | *brc-1(xoe4[null]); zim-1(tm1813)* | This study |
| JEL657 | *brd-1(xoe14[brd-1::gfp::3xFLAG])* | [1] |
| JEL1116 | *brc-1(xoe53[I23A])* *brd-1(xoe14[brd-1::gfp::3xFLAG])* | This study |
| JEL1117 | *brc-1(xoe52[triA]) brd-1(xoe14[brd-1::gfp::3xFLAG])* | This study |
| JEL515 | *brc-1(xoe7[gfp::3xFLAG::brc-1])* | [1] |
| JEL639 | *brc-1(xoe20[gfp::3xFLAG::brc-1I23A])* | This study |
| JEL1065 | *brc-1(xoe48[gfp::3xFLAG::brc-1triA])* | This study |
| JEL527 | *brc-1(xoe7[gfp::3xFLAG::brc-1]); syp-1(me17)/*  *nT1[unc-?(n754) let-? qls50] (IV;V)* | [1] |
| JEL1148 | *brc-1(xoe48[gfp::3xFLAG::brc-1triA]); syp-1(me17)/*  *nT1[unc-?(n754) let-? qls50] (IV;V)* | This study |
| JEL1274 | *brc-1(xoe7[gfp::3xFLAG::brc-1]); him-3(gk149) IV/nT1[qls51](IV;V)* | This study |
| JEL1281 | *brc-1(xoe48[gfp::3xFLAG::brc-1triA]); him-3(gk149) IV/nT1[qls51](IV;V)* | This study |
| JEL1285 | *brc-1(xoe7[gfp::3xFLAG::brc-1]); rec-8(ok978)/nT1 IV; coh-4(tm1857) coh-3(gk112) V/nT1 [qIs51] V* | This study |
| JEL1286 | *brc-1(xoe48[gfp::3xFLAG::brc-1triA]); rec-8(ok978)/nT1 IV; coh-4(tm1857) coh-3(gk112) V/nT1 [qIs51] V* | This study |
| JEL1162 | *brd-1(xoe18[null])* | This study |
| JEL1170 | *brd-1(xoe58[null-gfp::3xFLAG])* | This study |
| JEL1013 | *brc-1(xoe7[gfp::3xFLAG::brc-1]) brd-1(xoe18[null])* | This study |
| JEL1173 | *brc-1(xoe48[gfp::3xFLAG::brc-1(triA)]) brd-1(xoe18[null])* | This study |
| JEL846 | *brc-1(xoe34[mscarlet-I-GLO::3xFLAG::brc-1])* | This study |
| JEL1207 | *brc-1(xoe34[mscarlet-I-GLO::3xFLAG::brc-1]) brd-1(xoe18[null])* | This study |
| JEL1267 | *brc-1(xoe65[gfp(nd)::3xFLAG::brc-1])* | This study |
| JEL1277 | *brc-1(xoe65[gfp(nd)::3xFLAG::brc-1])* *brd-1(xoe18[null])* | This study |
| JEL1187 | *brd-1(xoe61[gfp::3xFLAG::brd-1])* | This study |
| JEL1189 | *brc-1(xoe4[null]) brd-1(xoe61[gfp::3xFLAG::brd-1])* | This study |
| JEL744 | *brc-1(xoe4[null]) brd-1(xoe14[brd-1::3xFLAG::gfp])* | [1] |

**References**

1. Li Q, Saito TT, Martinez-Garcia M, Deshong AJ, Nadarajan S, Lawrence KS, et al. The tumor suppressor BRCA1-BARD1 complex localizes to the synaptonemal complex and regulates recombination under meiotic dysfunction in *Caenorhabditis elegans*. PLoS Genet. 2018;14(11):e1007701.
2. Janisiw E, Dello Stritto MR, Jantsch V, Silva N. BRCA1-BARD1 associate with the synaptonemal complex and pro-crossover factors and influence RAD-51 dynamics during *Caenorhabditis elegans* meiosis. PLoS Genet. 2018;14(11):e1007653.
3. Bhalla N, Wynne, DJ, Jantsch V, Dernburg AF. ZHP-3 acts at crossovers to couple meiotic recombination with synaptonemal complex disassembly and bivalent formation in *C. elegans.* PLoS Genet. 2008;4(10):e1000235.
