# Supplementary material for: Differential requirement for BRCA1-BARD1 E3 ubiquitin ligase activity in DNA damage repair and meiosis in the Caenorhabditis elegans germ line
Source: PLoS Genet. 2023 Jan 30;19(1):e1010457. doi: 10.1371/journal.pgen.1010457 (PMC9910797; doi:10.1371/journal.pgen.1010457)
Supplement: S2 Table — (DOCX) [file pgen.1010457.s002.docx]

**Supplemental Table 2. CRISPR alleles**

| ***brc-1(xoe20[gfp::3xflag::brc-1(I23A)])III*** |
| --- |
| Guide: AGATGGCAGATGTTGCACTG |
| Repair template:  Homology arms were inserted into pDD282 as described for *gfp::3xflag::brc-1*(Li 2018) and I23A mutation was additionally introduced in 3’ homology arm using Q5 site-directed mutagenesis.  Primers for incorporating I23A mutation in 3’ homology arm:  Fwd: GAAATGTGGAGCTTGGTAATTTATTTTCAAAAAATC  Rev: AGTTCTTTTTGCAGTCGTG |
| Genotyping primers:  Fwd: TTTGTTTGAACGGAACCTTGC  Rev: AATAGCCCAAAAAGGCCGAA  2496bps from knock-in animals and 1436bps from wild type. |

| ***brc-1(xoe60[brc-1(I23A)])III*** |
| --- |
| Guide: AAGAACTGAAATGTGGAATT |
| Repair template:  ATGGCAGATGTTGCACTGAGGATCACAGAAACAGTGGCACGACTGCAAAAAGAACTGAAGTGCGGGGCCTGGTAATTTATTTTCAAAAAATCAACAAAAAAACCTTAAATTGGTTTTTTTTCAGCTGTTC |
| Genotyping primers:  Fwd: AGCGCGGGAGTTTTGAAAT  Rev: CCAGCGGCATTTGACTTTCC  Product 496bps.  FauI restriction digest  PCR product from correctly edited worms will be cut, resulting in 152 +344 bps.  Wild type sequence will not be cut. |

| ***brc-1(xoe48[gfp::3xflag::brc-1(I23A, I59A, R61A)]III*** |
| --- |
| Note: this was built by adding I59A R61A into *brc-1(xoe20[gfp::3xflag::brc-1(I23A)])III* |
| Guide: CCAGTACACTTCTACAAATT |
| Repair template:  TTTGATAAGTATCTCGACAACTTCGCTTATCCAGTACTGAAGCACATGCTGGGCATTGAACTTTTCGTTTTCGTTCGAAGCAAGCG |
| Genotyping primers:  Fwd: CACAGAAACAGTGGCACGAC  Rev: CAAAATAGCCCAAAAAGGCCGA  Product 873bps.  PspFI restriction digest  PCR product from correctly edited worms will be cut, resulting in 204 bps + 669bps products.  Wild type will not be cut. |

| ***brc-1(xoe62[brc-1(I23A, I59A, R61A)])III*** |
| --- |
| Note: this was built by adding I59A R61A into *brc-1(xoe60[brc-1(I23A)])III* |
| Guide: CCAGTACACTTCTACAAATT |
| Repair template:  TTTGATAAGTATCTCGACAACTTCGCTTATCCAGTACTGAAGCACATGCTGGGCATTGAACTTTTCGTTTTCGTTCGAAGCAAGCG |
| Genotyping primers:  Fwd: CACAGAAACAGTGGCACGAC  Rev: CAAAATAGCCCAAAAAGGCCGA  Product 873bps.  PspFI restriction digest  PCR product from correctly edited worms will be cut, resulting in 204 bps + 669bps products.  Wild type will not be cut. |

| ***brd-1(xoe18[brd-1(null)])III*** |
| --- |
| Guide: AGGGGAGACTTGCAATATCT |
| Repair template:  GATTTTGCCTCTTAAATAAAATTTCAGCAAAAAACCGAGGGGAGACTTGCAATAGGGAAGTTTGTCCAGAGCAGAGGTGACTAAGTGATAAGCTAGCTCTCGGATCATCTTGCAAACATGCTTATTGCTGGTAGGTATTGCAACC |
| Genotyping primers:  Fwd: ACGTTTCGAACAGCTATCGAGT  Rev: GCGATACATTCCCTGAACATAA  239bps from knock-in animals and 196bps from wild type. |

| ***brc-1(xoe34[mScarlet-i-glo::3xflag::brc-1])III*** |
| --- |
| **Guide: AGATGGCAGATGTTGCACTG** |
| Repair template:  GFP sequence in pDD282 was replaced by mScarlet-i-glo sequence obtained from plasmid pMS051(72) and homology arms were inserted into pDD282 as described for *gfp::3xflag::brc-1* (34). |
| Genotyping primers:  Fwd: TTTGTTTGAACGGAACCTTGC  Rev: AATAGCCCAAAAAGGCCGAA  2496bps from knock-in animals and 1436bps from wild type. |

| ***brc-1(xoe65[gfp(A206K, L221K, F223R)::3xflag::brc-1])*** |
| --- |
| Guide: AGATGGCAGATGTTGCACTG |
| Repair template:  A sequence containing GFP and homology arms at the N-terminus of BRC-1 was amplified using *gfp::3xflag::brc-1* genomic DNA as an original version of the repair template with the following primers.  Fwd: GCACCGACTGAAATATAGGCAC  Rev: TCGTTTTCGTTCGAAGCAAGC  This sequence was subcloned into a vector backbone using a Zero Blunt TOPO PCR cloning kit.  A206K, L221K, F223R mutations were introduced in GFP using Q5 site-directed mutagenesis kit with the following primers:  Fwd: CGTGACCACATGGTCCTCAAAGAGCGTGTCACCGCCGCCGGAATC  Rev: CTTCTCGTTTGGGTCCTTGGAGAGTTTGGATTGGGTGGAGAGGTAGTGG |
| Genotyping PCR:  Fwd: TTTGTTTGAACGGAACCTTGC  Rev: AATAGCCCAAAAAGGCCGAA  2496bps from knock-in animals and 1436bps from wild type. |

| ***brd-1(xoe61[gfp::3xflag::brd-1])*** |
| --- |
| Guide1: ATATCGAGTAATTCACTTGG  Guide2: CAGCTATCGAGTGTGTTAAA |
| REPAIR TEMPLATE:  TTGAGCTCGTTATCGGATAATTCCTAAAAAAATAACACTTTAATTAGGCATTTATTTCAGCTACTTTTTTCCTACCTCTTGTTTGCTTGAAAGCTGTCTAACTGCTCCTTTTCTGACTAATGGATCGAGCTCAGCAAGCTTTGAAATAATGTCTAAAAGCTCTGACGGCGGTCTCATGCTTAAAACTTAAAATTATAATTAAAAAGAGAATAAATTGACGAGAGGATCATTCTCGTGAATTTTCCAGACCAAGTAAACTGTATTTTTCTATAAAACACAAAAAATAAATGTAATTTTAGTTTAATGAAAGAAAAAGAAATAGAAGTAAAATATTTTGTTCGTAAAATAAAATGTATTCTGTGCGCACGCACCACACCTGACGCGCAAACAAACAAGTGCGATTTTCGAATTTTATTCTTTTCCTGTTTGATTTAATTATTGTTTCAGCGTCAAATAATGAGTAAAGGAGAAGAACTCTTTACCGGAGTCGTCCCAATTCTCGTCGAGCTCGACGGAGACGTAAACGGACACAAATTCTCGGTTTCCGGAGAAGGAGAAGGAGATGCTACTTATGGAAAACTCACCCTTAAATTCATTTGCACCACCGGAAAGCTCCCAGTGCCGTGGGTAAGTTTGTGATAATCCAATTTCAATTCGCAATGGTCATCGTTTTTTCAGCCGACCCTTGTGACTACATTCTGCTACGGAGTTCAATGCTTTTCCCGCTACCCAGACCACATGAAGCGTCACGATTTCTTCAAATCAGCCATGCCAGAAGGATACGTCCAGGAGCGAACAATTTTCTTCAAGGACGATGGAAACTACAAGACTCGTAAGTTTTTACTCCGCTTTTAACAATGGTTGTTTGACATCATTTTTTCAGGTGCTGAAGTCAAGTTTGAAGGAGATACTCTCGTGAACCGCATTGAGCTCAAGGGAATCGACTTCAAAGAAGATGGAAACATTCTTGGACACAAGCTCGAATACAACTATAACTCGCACAACGTGTATATCATGGCCGACAAGCAAAAAAATGGAATCAAGGTCGTAAGTTTGATGAAACGGTTTCGTCTTATATACACTAATGGTACTTTTCAGAACTTCAAGATTCGCCACAACATCGAAGACGGGTCGGTTCAACTCGCTGATCACTACCAGCAGAACACACCAATTGGAGATGGACCAGTCCTCCTCCCTGATAATCACTACCTTTCCACTCAATCCGCTCTTAGCAAGGATCCAAATGAGAAAAGAGATCACATGGTTCTTCTCGAGTTTGTCACCGCCGCCGGAATCACCCACGGAATGGACGAGTTGTACAAGGATTATAAAGACGATGACGATAAGCGTGACTACAAGGACGACGACGACAAGCGTGATTACAAGGATGACGATGACAAGAGAGGAGCATCGGGAGCCTCAGGAGCATCGTTTGAAAACACTAAAAAAGCATTGGAAACGTTTCGAACTGCAATTGAATGCGTGAAGTGGTAAGTTGTTGTACAAGATTTTGCCTCTTAAATAAAATTTCAGCAAAAAACCGAGGGGAGACTTGCAATATCTCGGATCATCTTGCAAACATGCTTATTGCTGGTAGGTATTGCAACCTAATAAATTGTCAATAACTTCAGTTTATGT |
| Genotyping PCR:  Fwd: TTCCGGTGTGAACTGAGAGC  Rev: AGAGCGTTTTCCAGACGGTT  2220bps from knock-in animals and 582bps from wild type. |
