# Supplementary material for: Differential requirement for BRCA1-BARD1 E3 ubiquitin ligase activity in DNA damage repair and meiosis in the Caenorhabditis elegans germ line
Source: PLoS Genet. 2023 Jan 30;19(1):e1010457. doi: 10.1371/journal.pgen.1010457 (PMC9910797; doi:10.1371/journal.pgen.1010457)
Supplement: S3 Table — (DOCX) [file pgen.1010457.s003.docx]

**Supplemental Table 3: RAD-51 statistical analyses**

**A. Avg # RAD-51 foci: zone 1**

| One-way ANOVA: p <0.0001  Tukey's multiple comparisons test | Adjusted P Value |
| --- | --- |
| *zim-1 vs. brc-1(I23A); zim-1* | 0.7224 |
| *zim-1 vs. brc-1(triA); zim-1* | 0.0116 |
| *zim-1 vs. brc-1(null); zim-1* | 0.9078 |
| *brc-1(I23A); zim-1 vs. brc-1(triA); zim-1* | 0.1086 |
| *brc-1(I23A); zim-1 vs. brc-1(null); zim-1* | 0.9868 |
| *brc-1(triA); zim-1 vs. brc-1(null); zim-1* | 0.0763 |

**B. Avg # RAD-51 foci: zone 2**

| One-way ANOVA: p <0.0001  Tukey's multiple comparisons test | Adjusted P Value |
| --- | --- |
| *zim-1 vs. brc-1(I23A); zim-1* | 0.0200 |
| *zim-1 vs. brc-1(triA); zim-1* | 0.0001 |
| *zim-1 vs. brc-1(null); zim-1* | <0.0001 |
| *brc-1(I23A); zim-1 vs. brc-1(triA); zim-1* | 0.2460 |
| *brc-1(I23A); zim-1 vs. brc-1(null); zim-1* | 0.0945 |
| *brc-1(triA); zim-1 vs. brc-1(null); zim-1* | 0.8835 |

**C. Avg # RAD-51 foci: zone 3**

| One-way ANOVA: p <0.0001  Tukey's multiple comparisons test | Adjusted P Value |
| --- | --- |
| *zim-1 vs. brc-1(I23A); zim-1* | 0.0007 |
| *zim-1 vs. brc-1(triA); zim-1* | <0.0001 |
| *zim-1 vs. brc-1(null); zim-1* | <0.0001 |
| *brc-1(I23A); zim-1 vs. brc-1(triA); zim-1* | <0.0001 |
| *brc-1(I23A); zim-1 vs. brc-1(null); zim-1* | <0.0001 |
| *brc-1(triA); zim-1 vs. brc-1(null); zim-1* | <0.0001 |

**D. Avg # RAD-51 foci: zone 4**

| One-way ANOVA: p <0.0001  Tukey's multiple comparisons test | Adjusted P Value |
| --- | --- |
| *zim-1 vs. brc-1(I23A); zim-1* | <0.0001 |
| *zim-1 vs. brc-1(triA); zim-1* | <0.0001 |
| *zim-1 vs. brc-1(null); zim-1* | <0.0001 |
| *brc-1(I23A); zim-1 vs. brc-1(triA); zim-1* | 0.0052 |
| *brc-1(I23A); zim-1 vs. brc-1(null); zim-1* | 0.2478 |
| *brc-1(triA); zim-1 vs. brc-1(null); zim-1* | <0.0001 |

**E. Avg RAD-51 foci pixel intensity: pre**

| One-way ANOVA: p <0.0001  Tukey's multiple comparisons test | Adjusted P Value |
| --- | --- |
| *zim-1 vs. brc-1(I23A); zim-1* | 0.3809 |
| *zim-1 vs. brc-1(triA) zim-1* | 0.9810 |
| *zim-1 vs. brc-1(null) zim1* | 0.9048 |
| *brc-1(I23A); zim-1 vs. brc-1(triA) zim-1* | 0.2265 |
| *brc-1(I23A); zim-1 vs. brc-1(null); zim1* | 0.9443 |
| *brc-1(triA); zim-1 vs. brc-1(null); zim1* | 0.7695 |

**F. Avg RAD-51 foci pixel intensity: in**

| One-way ANOVA: p <0.0001  Tukey's multiple comparisons test | Adjusted P Value |
| --- | --- |
| *zim-1 vs. brc-1(I23A); zim-1* | <0.0001 |
| *zim-1 vs. brc-1(triA) zim-1* | <0.0001 |
| *zim-1 vs. brc-1(null) zim1* | <0.0001 |
| *brc-1(I23A); zim-1 vs. brc-1(triA) zim-1* | <0.0001 |
| *brc-1(I23A); zim-1 vs. brc-1(null); zim1* | <0.0001 |
| *brc-1(triA); zim-1 vs. brc-1(null); zim1* | 0.9999 |

**G. Avg RAD-51 foci pixel intensity: post**

| One-way ANOVA: p <0.0001  Tukey's multiple comparisons test | Adjusted P Value |
| --- | --- |
| *zim-1 vs. brc-1(I23A); zim-1* | 0.4851 |
| *zim-1 vs. brc-1(triA) zim-1* | <0.0001 |
| *zim-1 vs. brc-1(null) zim1* | 0.3038 |
| *brc-1(I23A); zim-1 vs. brc-1(triA) zim-1* | 0.0010 |
| *brc-1(I23A); zim-1 vs. brc-1(null); zim1* | 0.9711 |
| *brc-1(triA); zim-1 vs. brc-1(null); zim1* | 0.0099 |
