# Supplementary figures and images for: Differential requirement for BRCA1-BARD1 E3 ubiquitin ligase activity in DNA damage repair and meiosis in the Caenorhabditis elegans germ line
Source: PLoS Genet. 2023 Jan 30;19(1):e1010457. doi: 10.1371/journal.pgen.1010457 (PMC9910797; doi:10.1371/journal.pgen.1010457)

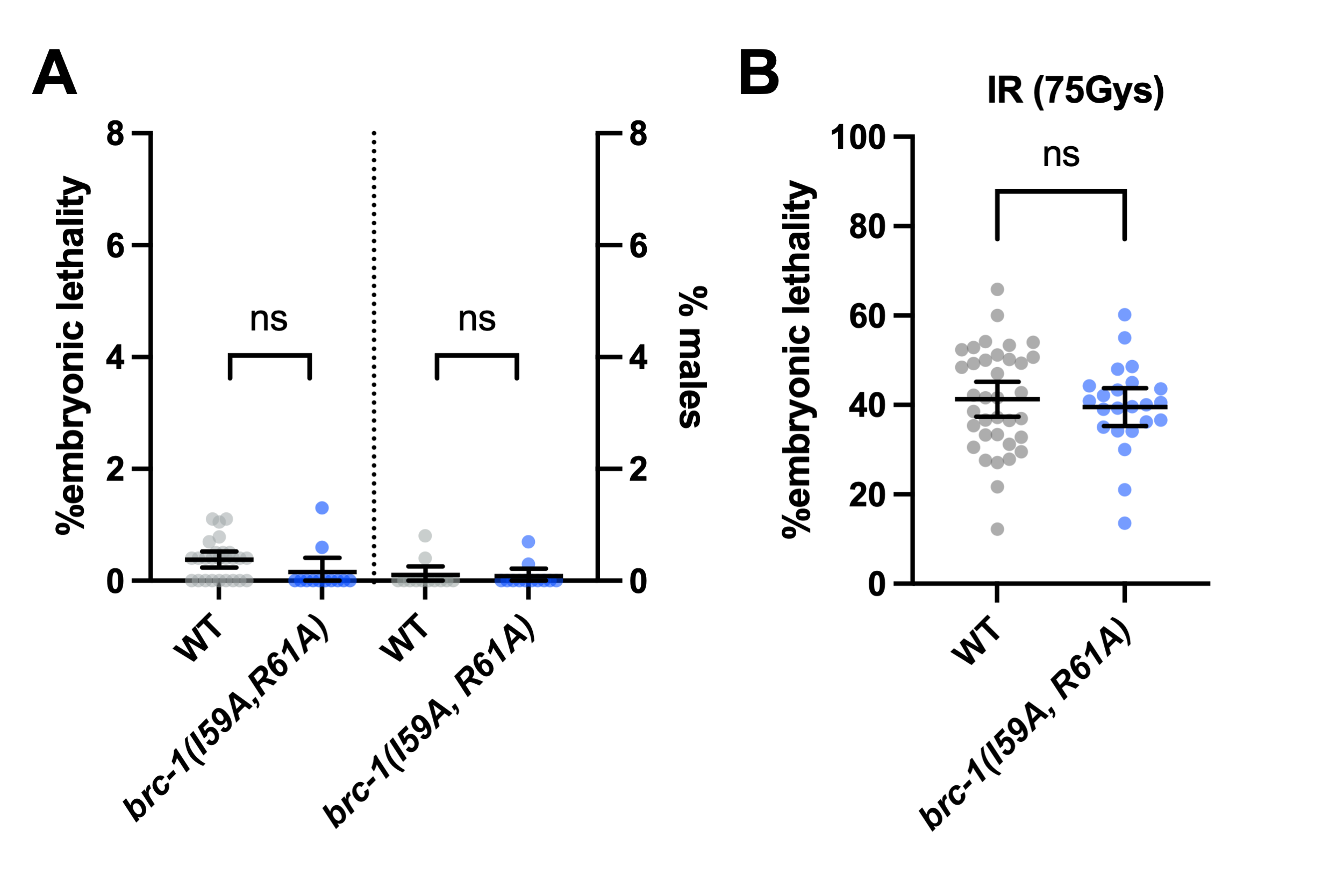

Supplement: S1 Fig — (A) Embryonic lethality (left Y axis) and male self-progeny (right Y axis) of wild type and brc-1(I59A, R61A) worms; n = 12, except for wild type embryonic lethality, n = 26. (B) Embryonic lethality in the presence of 75Gys IR of wild type (n = 36) and brc-1(I59A, R61A) (n = 23) worms. ns = not significant Mann-Whitney. (TIF) [file pgen.1010457.s004.tif]

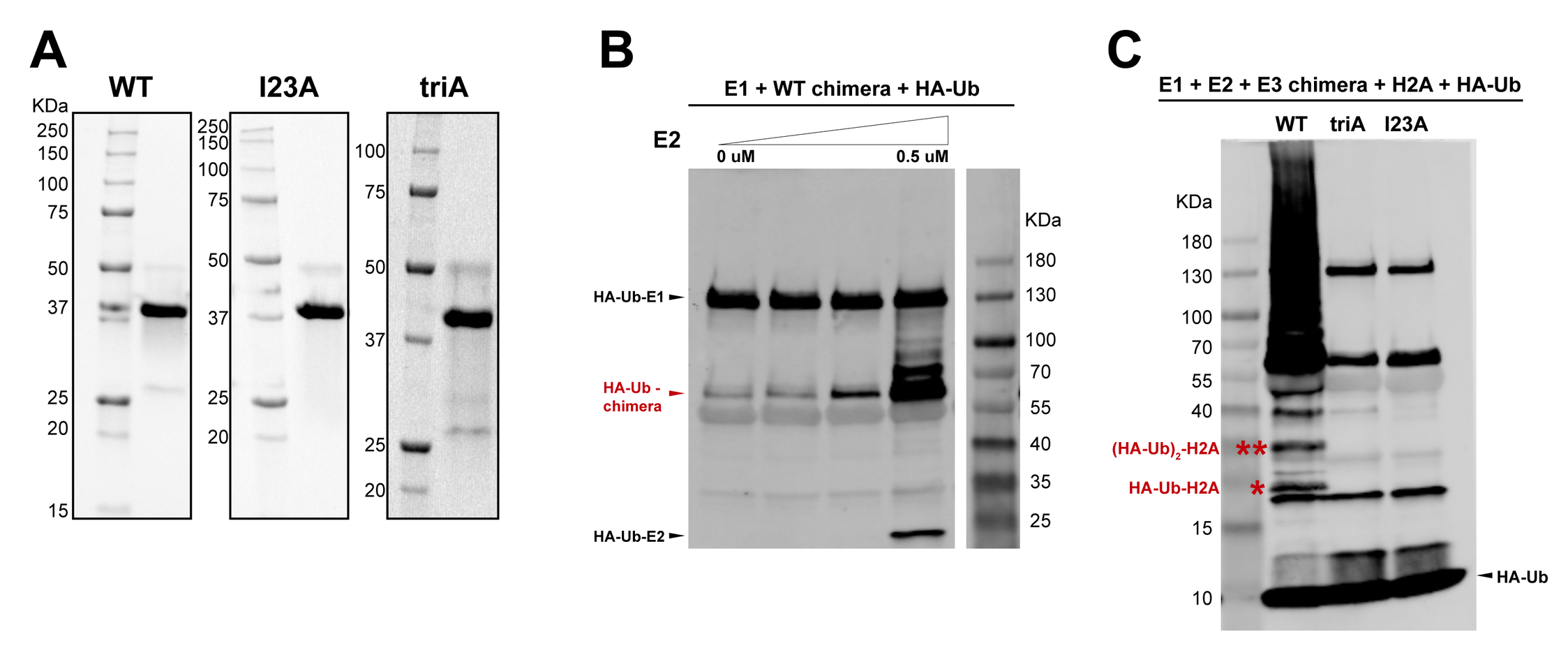

Supplement: S2 Fig — (A) Purified chimera proteins visualized on stain-free gels (proteins do not run true to size as they were loaded on gel in sample buffer without heat denaturation) with indicated molecular weight markers in kDaltons. (B) Titration of E2 conjugating enzyme in E3 ubiquitin ligase assay shows a non-specific mono-ub conjugate product in the absence of E2 enzyme. (C) Incorporation of mono- and di-HA-ubiquitin into histone H2A as visualized by antibody against HA. (TIF) [file pgen.1010457.s005.tif]

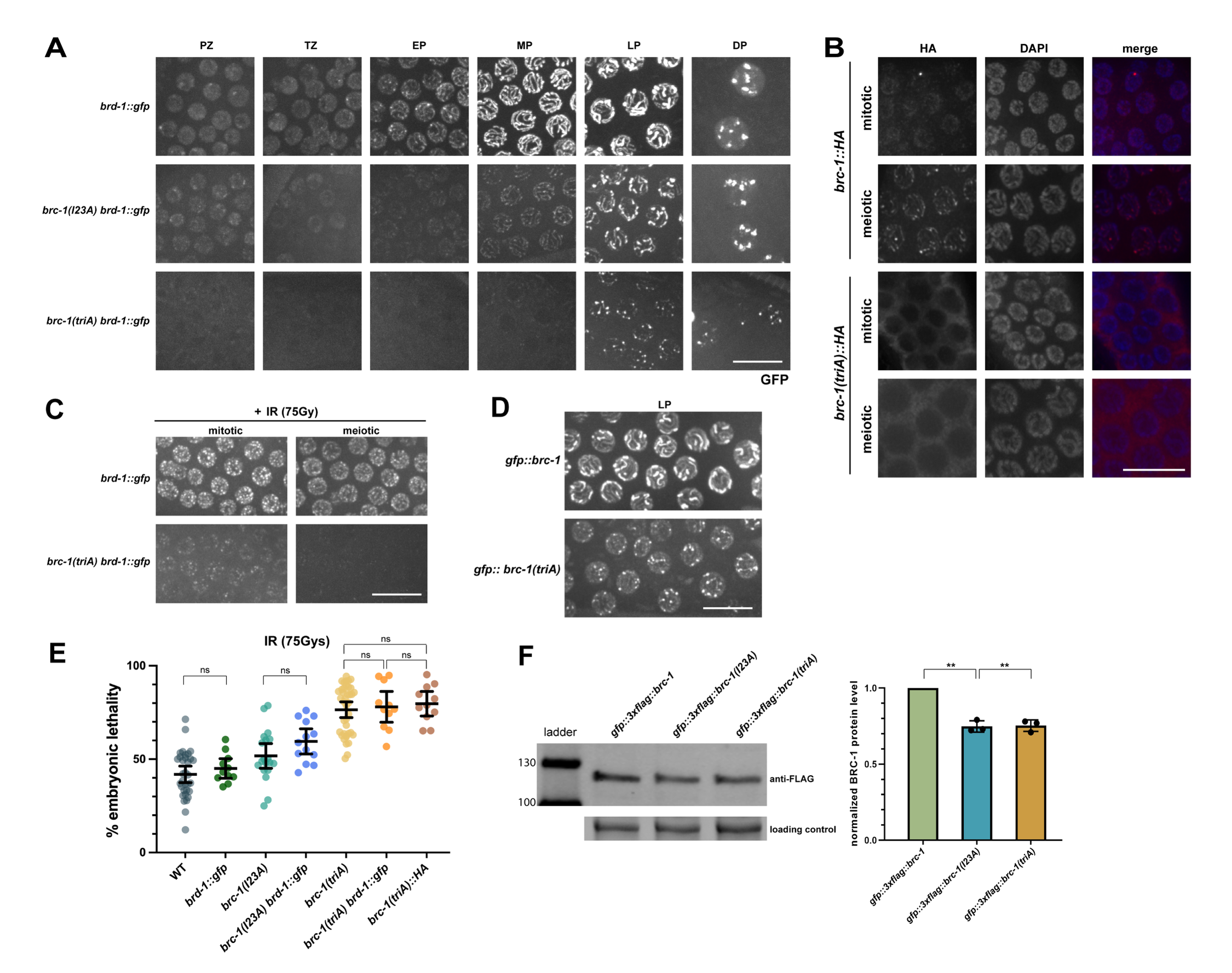

Supplement: S3 Fig — (A) BRD-1 protein localization shown by direct GFP fluorescence in live wild-type and mutant brc-1 worms in respective germ line regions. PZ = proliferative zone; TZ = transition zone; EP = early pachytene; MP = mid pachytene; LP = late pachytene; DP = diplotene. Scale bar = 10μm. (B) Immunostaining of BRC-1::HA and BRC-1triA::HA (red in merge) in fixed mitotic and meiotic (mid-pachytene) nuclei counterstained with DAPI (blue in merge). Scale bar = 10μm. (C) BRD-1::GFP and brc-1(triA) BRD-1::GFP fluorescence in live worms in mitotic and meiotic regions (mid-pachytene) in the presence of 75Gys IR. (D) GFP::BRC-1 and GFP::BRC-1triA fluorescence in live worms at late pachytene (LP) showing association with SC and crossover sites. (E) Embryonic lethality of worms treated with 75Gys IR. C-terminal GFP fusion to BRD-1 did not rescue viability in the brc-1 mutants. (F) Immunoblot (left) showing steady state levels of BRC-1 proteins from wild-type and mutant whole worm extracts. Levels of mutant BRC-1 proteins normalized to wild type protein from three independent experiments (right). ** p<0.01. (TIF) [file pgen.1010457.s006.tif]

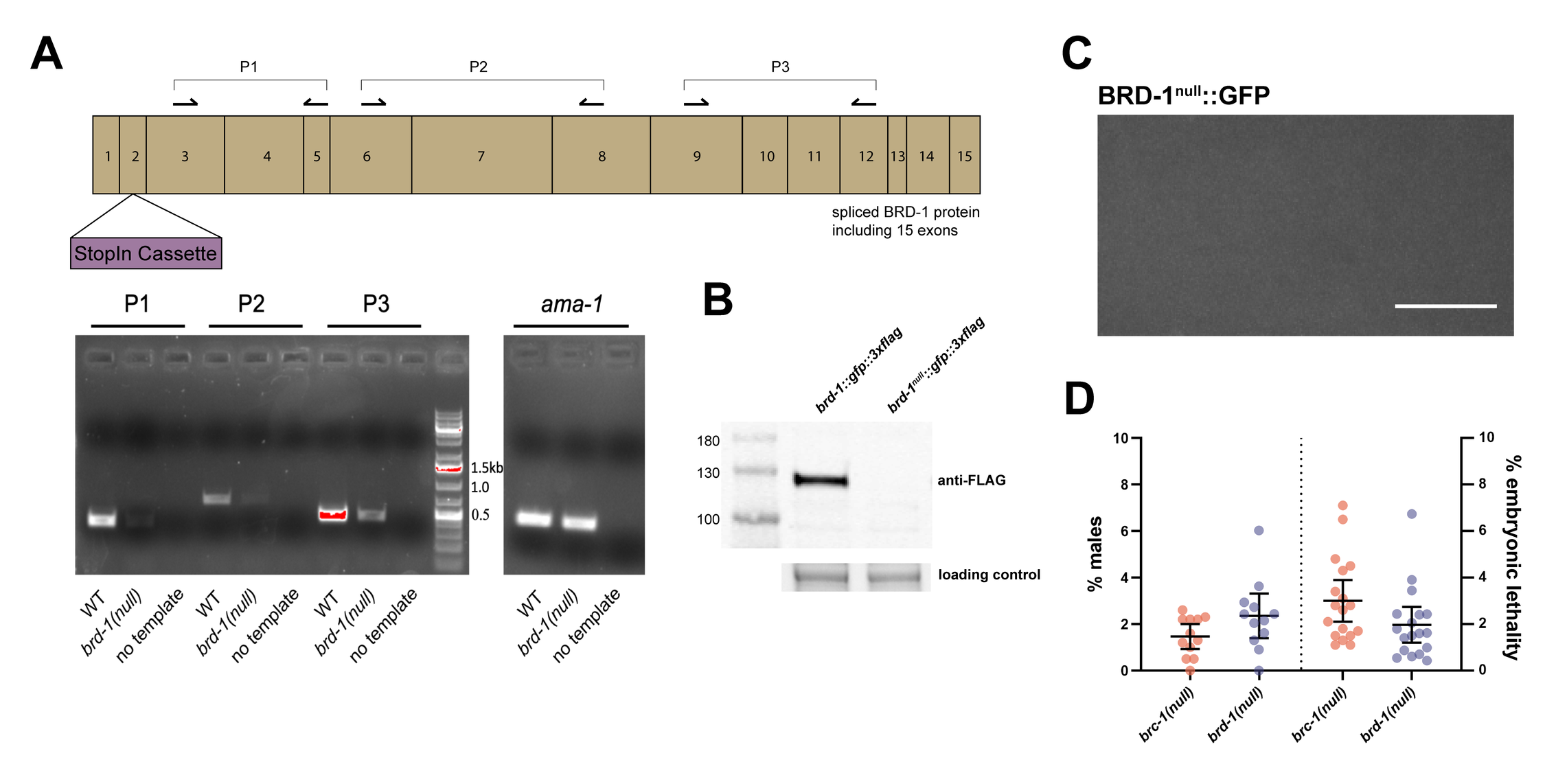

Supplement: S4 Fig — (A) BRD-1 exon structure and position of insertion of the stopin cassette. Primer pairs (P1-P3) used for RT-PCR of wild type and brd-1(null) cDNA are indicated. P1 Forward: cgccacatttcaacagaaacc, P1 Reverse: gcttctttgctgtagtcgtg; P2 Forward: cgcgtaattcgacaaaacgc, P2 Reverse: gcattaataactgcacccgc; P3 Forward: ggctcaacattagaaacaacgc, P3 Reverse: gatcaataatgcacgctctcag. ama-1 was used as control [95]. (B) Immunoblot of whole worm extracts of BRD-1::GFP::3xFLAG and BRD-1null::GFP::3xFLAG with indicated molecular weight markers. (C) No GFP fluorescence was observed in brd-1(null)::gfp worms. Scale bar = 10μm. (D) Male self-progeny (left Y axis; n = 12) and embryonic lethality (right Y axis; n = 18) of brc-1(null) and brd-1(null) worms. No statistical differences were observed by Mann-Whitney. (TIF) [file pgen.1010457.s007.tif]

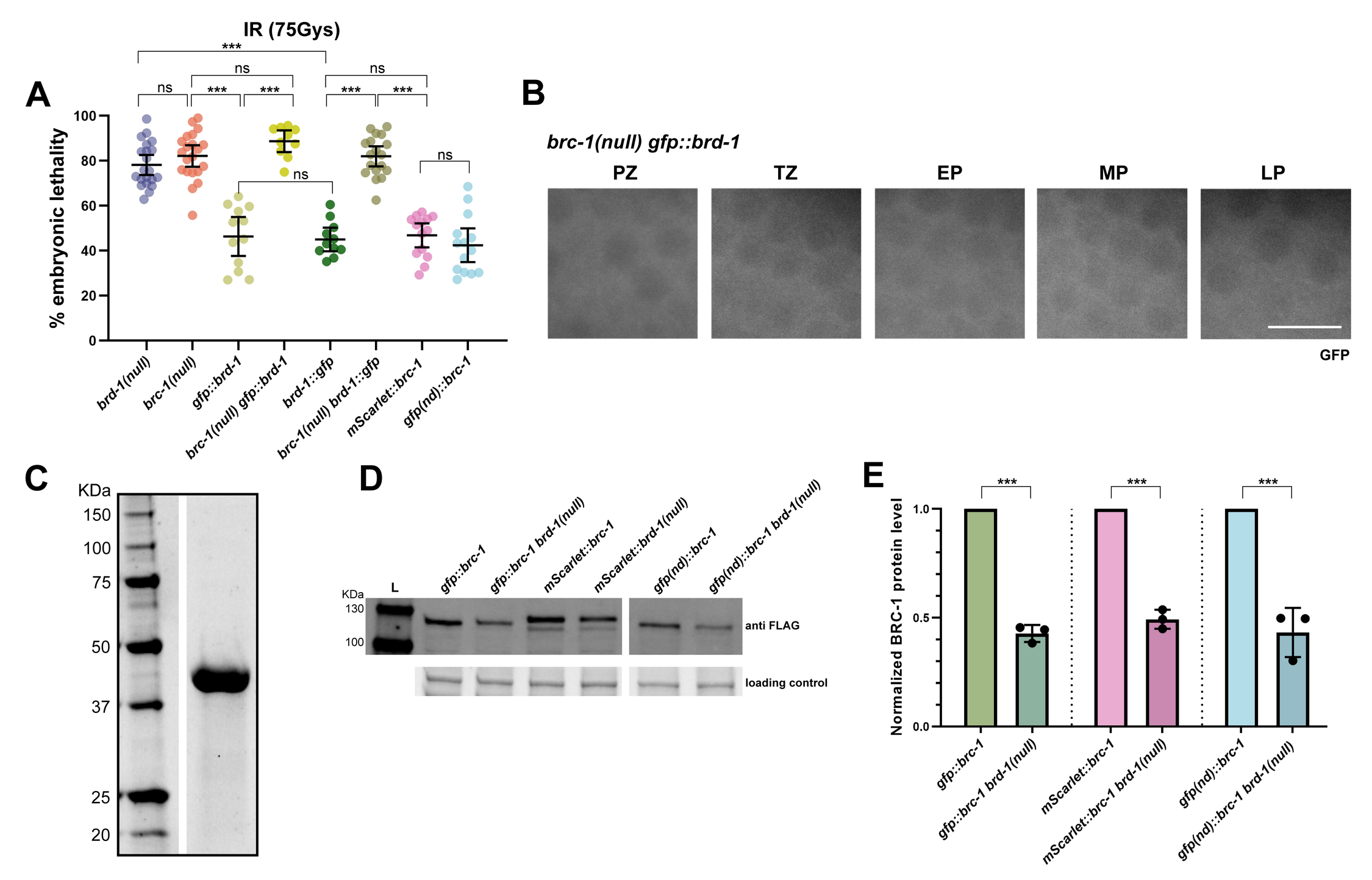

Supplement: S5 Fig — (A) Embryonic lethality in the presence of 75Gys IR was examined in brd-1(null) (n = 21), brc-1(null) (n = 21), gfp::brd-1 (n = 12), brc-1(null) gfp::brd-1 (n = 10), brd-1::gfp (n = 11), brc-1(null) brd-1::gfp (n = 18), mScarlet::brc-1 (n = 14), gfp(nd)::brc-1 (n = 14). (B) Image of GFP::BRD-1 fluorescence in the brc-1(null) mutant. Scale bar = 5μm. (C) E. coli purified GFP::BRC-1 RING protein visualized on a stain-free gel with indicated molecular weight markers. (D) Immunoblot of GFP::BRC-1, mScarlet::BRC-1, and GFPnd::BRC-1 in the brd-1(null) mutant. (E) Quantification of relative steady state levels of GFP::BRC-1, mScarlet::BRC-1, and GFPnd::BRC-1 in the brd-1(null) mutant. (TIF) [file pgen.1010457.s008.tif]
